# Neuroimaging study of electroconvulsive therapy for depression

**DOI:** 10.3389/fpsyt.2023.1170625

**Published:** 2023-06-09

**Authors:** Xiaolu Chen, Hanjie Yang, Long-Biao Cui, Xiao Li

**Affiliations:** ^1^The First Branch, The First Affiliated Hospital of Chongqing Medical University, Chongqing, China; ^2^Department of Neurology, The Thirteenth People’s Hospital of Chongqing, Chongqing, China; ^3^Department of Radiology, The First Affiliated Hospital of Xi’an Jiaotong University, Xi’an, China; ^4^Schizophrenia Imaging Lab, Fourth Military Medical University, Xi’an, China; ^5^Department of Psychiatry, The First Affiliated Hospital of Chongqing Medical University, Chongqing, China

**Keywords:** depression, electroconvulsive therapy, magnetic resonance imaging, positron emission tomography, mechanism, arterial spin labeling

## Abstract

Electroconvulsive therapy (ECT) is an important treatment for depression. Although it is known as the most effective acute treatment for severe mood disorders, its therapeutic mechanism is still unclear. With the rapid development of neuroimaging technology, various neuroimaging techniques have been available to explore the alterations of the brain by ECT, such as structural magnetic resonance imaging, functional magnetic resonance imaging, magnetic resonance spectroscopy, positron emission tomography, single photon emission computed tomography, arterial spin labeling, etc. This article reviews studies in neuroimaging on ECT for depression. These findings suggest that the neurobiological mechanism of ECT may regulate the brain functional activity, and neural structural plasticity, as well as balance the brain’s neurotransmitters, which finally achieves a therapeutic effect.

## Introduction

Depression is characterized by persistent low mood and reduced volitional activity, with high incidence, recurrence, and suicide rates, which seriously endanger human physical and mental health (1). According to reports, more than 350 million people worldwide suffer from depression (2). Therefore, it is of great importance to understand the causes of depression and how to combat it. Neuroimaging is now widely used to investigate the pathogenesis of depression, and studies have found that structural or functional alterations in the prefrontal cortex, hippocampus, amygdala, limbic system, and other regions of the brain are significantly associated with the onset of depression.

Electroconvulsive therapy (ECT) is a treatment for mental illness by passing a certain amount of electric current through the brain, causing epileptiform discharges. Since ECT was invented by Italian scientists in 1938, it has now been used in the treatment of mental disorders for more than 80 years (3). As the knowledge of ECT has gradually increased, it has also been found to have good effects on the improvement of depressive symptoms, and the general opinion is now that ECT is one of the most effective methods for depression (4) and has been widely used for severe depressive disorders (5). Some studies have reported that ECT can provide up to 70%–80% remission rate of depressive symptoms (6, 7). Also, its benefits include reduced patient hospitalization rates, and reduced risk of suicide (8–10).

Even though ECT has remarkable efficacy, but how long does ECT affect the brain is unclear, for example, the ECT induced memory loss could last for 2–3 months (11), but some study reported that some still had loss of memory even 3 years after ECT (12). More and more neuroimaging studies are used to understand the effect of ECT. Neuroimaging techniques, as a means of noninvasive observation of brain alterations, can be used to find markers of pathophysiological processes in psychiatric disorders. Previous studies on neuroimaging have found that patients with depression may experience structural or functional abnormalities (13, 14). Neuroimaging studies of depressed patients before/after ECT have also found that ECT can alter the structure and function of specific brain regions. For example, studies have found that ECT can cause changes in hippocampal volume (15, 16). Although there are more and more brain imaging studies trying to explore the therapeutic mechanisms of ECT, there are inconsistencies in the brain regions that cause alterations, so we try to review the brain imaging studies on ECT for depression.

### Search strategy

References included in the study were identified through multiple searches of the Embase and PubMed/MEDLINE for articles published until December 2022, using combinations of the following search terms: “Depression”, “Major depressive disorder”, “Gray matter volume”, “GMV”, “Cortical thickness”, “White matter”, “Functional magnetic resonance imaging”, “fMRI”, “Regional homogeneity”, “ReHo”, “Amplitude of low frequency fluctuations”, “ALFF’, “Functional connectivity”, “FC’, “Magnetic Resonance Spectroscopy”, “MRS’, “Positron Emission Tomography”, “PET’, “Single photon emission computed tomography”, “SPECT”, “Arterial spin labeling”, “ASL”, “electroconvulsive therapy” and “ECT”. Due to the breadth of relevant studies, published reviews, meta-analyses, randomised controlled trials and recent studies were prioritised to provide a comprehensive and up-to-date overview of research on ECT in patients with depression.

## Structural magnetic resonance imaging

### Gray matter volume

Previous studies have found that ECT can increase brain gray matter volumes of depressed patients commonly in the hippocampus, amygdala, and temporal lobe (17–19). Among all studies, the most consistent trend of altered brain structures after ECT is to increase the volume of the temporal lobe as well as subcortical areas such as the hippocampus, amygdala, striatum, and anterior cingulate gyrus (20). Some researchers have explored the relationship between ECT and depression, such as Tendolkar et al. (17) found ECT could increase the volumes of bilaterally hippocampus and amygdala, and HAMD scores as well as depressive symptoms significantly reduced after ECT. Gryglewski et al. (21) also found structural changes in hippocampal subregions and amygdala after ECT, and these structural changes were closely related to the pathophysiological mechanisms of depression and the development of stress-related disorders. Cao et al. (22) used the latest hippocampal segmentation method and found that ECT could increase the volume of corpus striatum, granule cell layer, molecular layer, and hypothalamus, while the efficacy of ECT for each patient could be accurately predicted. Jorgensen et al. (23) focused on the volume of hippocampus, amygdala, dorsolateral prefrontal cortex (DLPFC), orbitofrontal cortex, and hypothalamus and found the volume of hippocampus and amygdala in patients increased significantly after ECT, while the volume of DLPFC decreased slightly, but due to the lack of correlation between changes in these brain regions and antidepressant effects, the remodeling of these brain structures cannot directly respond to the antidepressant mechanisms of ECT. There are also some studies based on voxel-based morphometry (VBM) to explore whole brain structural abnormalities, such as Ota et al. (24) found that depressed patients had a significant increase in the volume of bilateral inferior temporal cortex, and the right anterior cingulate gyrus after ECT, and the increased volume correlated with clinical improvement. Sartorius et al. (25) analyzed the changes in structural magnetic resonance pre/post-ECT, found a more pronounced increase in gray matter volume in the bilateral temporal lobe, middle cingulate gyrus, insula, and the putamen. Some studies even found that after unilateral ECT, only the unilateral hippocampus, amygdala, insula, and subgenual cortex volumes increased (16, 26). Changes of hippocampus volume were concerned commonly after ECT, studies have shown a significant reduction in hippocampal volume in depressed patients (27–29). Tendolkar (17) found an increase in bilaterally hippocampal volume after ECT, and Abbott (30) found a significant increase of right hippocampal volume after ECT. All these studies suggest that increased hippocampal volume after ECT may be one of the potential antidepressant mechanisms. However, there are also different opinions, such as Gbyl et al. (31) who argued that current MRI studies have not paid enough attention to the hypothesis that ECT may lead to brain damage, and suggested that future studies should explore the relationship between brain volume changes and cognitive impairment. In addition to focusing on the therapeutic mechanism of ECT, there are also many studies that agree that the increase of hippocampal volume caused by ECT may be related to cognitive side effects, such as Argyelan et al. (32) suggested that hippocampal enlargements are associated with cognitive side effects. van der A et al. (33) found similar change, which is ECT-induced left hippocampal enlargement correlated with decreases in verbal memory functioning. Gbyl et al. (34) found ECT-related transient increases in the volume of major hippocampal subregions within-patients are associated with memory impairment, based on previous studies, ECT-induced volume changes can be due to vascularization (35), inflammation (36) or vasogenic oedema (37) and/or neuroplastic mechanisms including neurogenesis (38), synaptogenesis (39) and gliogenesis (40), but further studies are still needed.

### Cortical thickness

Studies have suggested that cortical thickness varies in a variety of psychiatric disorders. For example, a study focusing on bipolar disorder found that the patient group had reduced cortical thickness in the left superior temporal gyrus, bilateral prefrontal compared with healthy controls (HCs) (41), and in a study of cortical thickness in depressed patients, it was found that cortical thickness of right middle frontal gyrus and left anterior cingulate cortex (ACC) were thickened in depressed patients compared with HCs (42). It has been demonstrated that ECT can change cortical thickness in different brain regions (e.g., superior temporal gyrus, inferior temporal gyrus, insula, anterior cingulate gyrus, prefrontal cortex, fusiform gyrus) (25, 43). van Eijndhoven et al. (44) evaluated 19 patients with refractory depression after ECT and found cortical thickness in the left temporal pole, left middle temporal lobe, and right insula increased significantly after ECT, and the average remission rate of depressive symptoms was 57%. A study with a 6-months follow-up after ECT, exploring the cortical thickness in 26 brain regions, showed that significant increased cortical thickness of frontal, temporal, and insula could be seen immediately after ECT, while after 6 months, the thickened cortical thickness returns to baseline (45). Some studies suggested that the cortical thickness of precentral gyrus, lateral occipital lobe can predict relapse rate after ECT for depression (46). Pirnia et al. (43) found that increased cortical thickness of bilateral ACC and superior temporal gyrus cortex after ECT, especially changes in ACC can predict the early efficacy of ECT.

### White matter

Alterations in the white matter of the brain are also the focus of brain science research, and diffusion tensor imaging (DTI) techniques have an important place in the study of white matter by assessing the diffuse activity of water molecules in neural tissue, which allows noninvasive probing of the direction and intensity of white matter trajectories. One study that included 2,937 psychiatric patients with depression, schizophrenia, bipolar disorder, or autism, focusing on changes in the white matter, found that patients with schizophrenia, bipolar disorder, or autism had similar structural changes in the corpus callosum, but the similarity change was not found in depression patients. The study also found that patients with schizophrenia and bipolar disorder had alterations in the limbic system compared with HCs, but not found in depressed patients (47). Chen et al. (48) conducted a meta-analysis of brain microstructural abnormalities in unmedicated depression patients by DTI, and a reduction of fractional anisotropy (FA) was found in bilateral anterior internal capsule, corpus callosum, right superior frontal gyrus, and right inferior temporal gyrus. Gbyl et al. (31) found ECT could increase the integrity of white matter pathways in the frontal lobe as well as temporal lobe, but the relationship between increased white matter volumes and treatment outcome could not been found. Yrondi et al. (49) used mean diffusivity (MD) to examine patients with refractory depression and found a reduction of MD in the hippocampus and left amygdala after ECT, and considered that ECT could modify microstructural integrity. Gryglewski et al. (50) tried to explore white matter changes in refractory depression patients taking unilateral ECT and found an increased axial diffusivity (AD) in the right posterior limb of internal capsule, but no correlation was found between changed AD and clinical effects. Repple et al. (51) analyzed changes in white matter structure before and after ECT in depressed patients and found increased MD in the right hemisphere after ECT. Kubicki et al. (52) found changed structural connectivity of hippocampal neural circuits after ECT. Lyden et al. (53) observed a significant increase of FA in the dorsal frontal-limbic circuits in depressed patients after ECT. A series of studies using DTI assessment suggest that ECT can effectively modulate white matter structure in depressed patients.

## Functional magnetic resonance imaging

### Resting-state functional magnetic resonance imaging

Blood oxygen level-dependent functional magnetic resonance imaging (BOLD-fMRI) has been applied in the field of brain function research since the 1990s and is now widely used as a non-invasive brain imaging assessment technique for psychiatric disorders. fMRI has the following advantages: non-invasive, non-radioactive, and reproducible. It can also analyze the dynamic activity of neurons on an individual and can analyze different response patterns between adjacent cortices. For spontaneous low-frequency activity acquired in the resting state, it is thought to reflect the spontaneous brain functional activity of the central nervous system in the resting state. Therefore, resting-state fMRI (rs-fMRI) has obvious clinical advantages. rs-fMRI is also particularly suitable for brain imaging studies of depressed patients, and the main analysis methods currently available are: regional homogeneity, amplitude of low frequency fluctuations, fractional amplitude of low frequency fluctuations, and functional connectivity.

### Regional homogeneity

Regional homogeneity (ReHo) responds to the degree of synchronization of neuronal activity in regional brain regions (54). It has been shown that the brain can have spontaneous neural activity with a high degree of synchrony in BOLD signals (55, 56). Studies on the relationship of depression and ReHo are common, such as Li et al. (57) found that, compared with HCs, depressed patients had lower ReHo in the precuneus. Kong et al. (58) found that there was a significant decreased ReHo in the bilateral superior frontal gyrus after ECT and a significant correlation was found between ReHo in the right superior frontal gyrus and changed HAMD before and after ECT. Mo et al. (59) found that after ECT, ReHo increased in left angular gyrus in depressed patients. Qiu et al. (60) also focused on the changed ReHo in depressed patients after ECT and found that patients showed increased ReHo in the bilateral frontal lobe, bilateral parietal lobe and right caudate nucleus after 8 sessions of ECT, while reduced ReHo was found in the left anterior cerebellar lobe, right cingulate gyrus, and right superior temporal gyrus. Argyelan et al. (61) found ReHo increase significantly in the left angular gyrus in refractory depression patients after ECT. Therefore, ReHo has the potential to be used as a response indicator for ECT used in depressed patients.

### Amplitude of low frequency fluctuations/fractional amplitude of low frequency fluctuations

Amplitude of low frequency fluctuations (ALFF) as well as fractional amplitude of low frequency fluctuations (fALFF) have been demonstrated valid in the assessment of spontaneous brain activity in different psychiatric disorders (62–64). Studies on depression and ALFF/fALFF involving different ages and different brain regions, such as studies showed abnormal ALFF in the cingulate gyrus in depressed patients (65, 66). Wang et al. (67) found ALFF in the right precuneus and posterior cingulate gyrus decreased significantly in depressed patients compared with HCs. A study focused on depression adolescents showed decreased ALFF in the left somatosensory cortex and increased ALFF in the left insula compared with HCs (68). ECT could change ALFF/fALFF in different brain regions, such as Kong et al. (58) found ALFF in the left middle frontal gyrus increased after ECT, while ALFF in the left middle cingulate gyrus, left precentral gyrus, right superior frontal gyrus, and right middle frontal gyrus decreased. Du et al. (69) found a significant decreased ALFF in the right posterior cerebellar lobe of depressed patients after ECT, and previous studies found that ECT could increase ALFF in the medial prefrontal, orbitofrontal, and perigenual ACC (pgACC) (70, 71). Argyelan et al. (61) compared the differences brain function between refractory depression patients and HCs, and found increased fALFF in the right cingulate cortex in the patient group at baseline, indicating brain function hyperactivity, and after ECT, fALFF in the cingulate gyrus decreased significantly, indicating that ECT could significantly improve abnormal brain functional activity.

### Functional connectivity

Functional connectivity (FC) is a method of calculating the similarity in time of blood oxygen signals between different brain regions. The signal of each brain region changes over time, and for a specific brain region, the sequence of its signal can be extracted from the MRI data, and the correlation of the time series between different anatomical regions of the brain can be obtained (72, 73). FC analysis methods have been widely used to explore the pathogenesis of depression, and analysis methods included: seed-based analysis, graph theory analysis, and brain network connectivity analysis, etc. Previous studies have shown that the hippocampus (74, 75), amygdala (76–78), thalamus (79, 80), insula (76, 81), and cingulate gyrus (74, 82), are related to emotional or cognitive processing in depressed patients. It was found that ECT could increase FC between hippocampus-occipitotemporal area, the dorsal lateral prefrontal-posterior cingulate gyrus, and the prefrontal-limbic system (75–83). Studies showed rs-FC changed in brain regions such as bilateral anterior cingulate gyrus, dorsomedial prefrontal cortex, bilateral superior frontal gyrus, left angular gyrus, left precuneus, bilateral hippocampus, right superior temporal gyrus, right insula, and cerebellum after ECT (84). Mo et al. (59) found FC increased significantly in the left angular gyrus, bilateral inferior temporal gyrus and bilateral middle frontal gyrus after ECT in depressed patients, accompanied by improvement in emotional symptoms. In FC study which used cingulate gyrus as a seed, found that the FC between left pgACC-left parahippocampal gyrus increased after ECT (82). Another study found that the FC of subcallosal cingulate cortex-bilateral hippocampus, subcallosal cingulate cortex-bilateral temporal poles and subcallosal cingulate cortex-ventral prefrontal cortex decreased after ECT (83). Some studies noted rs-FC of subcallosal cingulate cortex-amygdala and subcallosal cingulate cortex-fusiform gyrus changed after ECT. Leaver et al. (84) found that rs-FC of the left DLPFC-subcallosal cingulate cortex may predict the efficacy of ECT for depression.

Graph theory analysis is applied to complex network functions of human brain, which focuses on the relationship between nodes and edges. The application of graph theory in the FC analysis of the brain can be characterized by different metrics to demonstrate different aspects of connectivity. Such as (i) average path length; (ii) clustering coefficient; (iii) degree of node; (iv) centrality measures; (v) level of modularity, etc. The clustering coefficient reflects the local connectivity of the network. The node degree is the simplest metric to quantify the total number of connected nodes. A node with a higher degree indicates that it plays an important role in the information flow of a particular network. Path length is another graph theoretic metric that represents the global level of efficiency of the network. Thus, the shortest path length represents the minimum number of edges required to connect one node to another in the network. Degree centrality (DC) has been widely studied in depressed patients, some studies found that depressed patients with suicidal ideation had elevated DC in the inferior frontal gyrus, orbital part, the supplementary motor area compared with HCs (85, 86). Wagner et al. (87) found that reduced DC in the frontoparietal network can distinguish suicidal ideation and suicide attempts in depressed patients. A study on DC alterations after ECT, which focused on anhedonia in depressed patients, found that ECT significantly increased DC in bilateral dorsomedial prefrontal cortex, right DLPFC, and bilateral orbitofrontal cortex, and alterations of anhedonia were positively correlated with DC in the abovementioned regions (88).

Brain networks change in depressed patients are commonly observed, and a number of studies have also suggested that ECT may play a role by modulating brain networks in depressed patients. Wang et al. (89) observed the default mode network (DMN), dorsal attention network (DAN), executive control network (CON), salience network (SN), and sensory-motor network (SMN) and tried to explore the FC within each network and between networks, and found that within the network, the FC within CON of depressed patients increased after ECT, and between different networks, DMN-SN, CON-DMN, CON-DAN, and CON-SN increased after ECT. Pang et al. (90) also focused on changes of brain networks in depressed patients after ECT, and found FC within DMN, and FC of DMN-CEN significantly increased after ECT, and could predict the efficacy of depression.

### Magnetic resonance spectroscopy

Magnetic resonance spectroscopy (MRS) is used to identify abnormal metabolism by measuring changes in tissue concentrations of metabolites in humans and observing different peaks and ratios in the spectral profile. It is a non-invasive technique that measures specific functional areas of the brain and analyzes neuro-biochemicals. These compounds include γ-aminobutyric acid (GABA), glutamate (Glu), N-acetyl-L-aspartate (NAA), glutamine (Gln), creatine (Cr), etc.

Glu plays a key role in the pathophysiology of depression (91). There is evidence that the levels of Glu and Gln are reduced in pgACC (92, 93), whereas the levels of Glu in DLPFC are unchanged (94, 95). ECT induces changes in glutamatergic neurotransmitters that may be closely related to their antidepressant effects (96, 97). Njau et al. (98) found depressed patients treated with ECT showed that Glx (Glu and Gln) increased in the subgenual ACC (sgACC) but decreased in the left hippocampus, and these changes were correlated with clinical improvement. Some studies found elevated levels of Glx in the DLPFC and ACC after ECT (99, 100), but these results could not reproducible (96), Overall, the metabolism of Glu is an important component of ECT efficacy for depression, but the exact mechanism still needs further research. The reduction of GABA in cerebrospinal fluid and the frontal cortex has been frequently reported in patients with depression (101). Meanwhile, some studies have confirmed an increased GABA in serum levels and occipital lobe after ECT (102, 103). However, Knudsen et al. (104) measured changes of GABA in the prefrontal and occipital cortex pre/post-ECT and found compared with HCs, no significant differences were detected by GABA/Cr levels in the prefrontal or occipital lobes, while the patient group did not show significant differences in GABA, Glu and Gln after ECT, so it was speculated that GABA alterations should not be considered as a key factor of ECT for depression. NAA is a marker of neurons and axons, and it can reflect the number of neurons as well as their function. Proton MRS (H-MRS) showed that ECT could increase NAA levels in the ACC and amygdala, suggested that ECT may have a neuromodulation effect. Njau et al. (98) examined depressed patients who underwent ECT measured by 1H MRS and found decreased NAA in the left hippocampus of the patient group compared with HCs. At the same time, reduction of NAA in the dorsal ACC (dACC) and right hippocampus were found after ECT. Overall, the antidepressant effects of ECT may involve different neuro-transporter systems, and the mechanisms of neuro-transporter alterations between them are not fully understood, which is one of the reasons for the inconsistent findings.

### Positron emission tomography/single photon emission computed tomography

Positron emission tomography (PET) and Single photon emission computed tomography (SPECT) monitor local cerebral blood flow (CBF) by injecting radionuclides into the body and then imaging γ-rays emitted from the body. Studies on CBF with ECT are common, but the findings are inconsistent. Mervaala et al. (105) found ECT could reduce CBF of temporal lobe and bilateral parietal lobe by SPECT, while Milo et al. (106) suggested that increased CBF in frontal lobe was significant of ECT for antidepressant effects. However, Schmidt et al. (107) found ECT decreased brain metabolism in the anterior and posterior frontal lobes assessed by PET. Suwa et al. (108) also found that ECT could reduce brain metabolism in the frontal and lateral temporal lobes, and the reduced brain metabolism was correlated with the antidepressant effect of ECT (109). On the other hand, previous studies on PET probing neurotransmitter changes after ECT, Masuoka et al. (110) used [^18^F] FE-PE2I PET to detect neurotransmitter levels in depressed patients before, during and after ECT and found that all patients showed reduced dopamine transporters in the striatum, and the dopamine system has been shown to be one of the mechanisms of ECT. Tiger et al. (111) used PET and [^11^C] raclopride to detect neurotransmitter levels in patients with depression before and after ECT, as well as HCs, and found that depressed patients had decreased [^11^C] raclopride binding in all three striatal regions compared to HCs. Yatham et al. (112) used [^18^F] setoperone PET and found that depressed patients treated with ECT showed significant reduced 5-HT2 receptors in all cortical regions of the brain and the decreased 5-HT2 receptors in the right parahippocampal gyrus, right lingual gyrus and right medial superior frontal gyrus were correlated with improvements in depressive symptoms, this result is also consistent with previous studies on antidepressant drugs (113–115).

PET is also widely used to assess ECT-induced changes of [^18^F]-fluorodeoxyglucose (FDG). One of the most consistent findings was decreased glucose metabolism in the bilateral medial and inferior frontal regions, and increased glucose metabolism in the hippocampus, middle temporal lobe, left occipital lobe, and parietal lobe (116). Bak et al. (117) used [^18^F]-FDG PET to study the efficacy of ECT in a 55 years-old woman with late-onset depression and found that PET/CT images of the patient’s brain showed decreased brain metabolism, whereas after ECT, the PET imaging returned to normal brain metabolism. Hassama et al. (118) found decreased metabolic in the frontal, parietal and temporal cortices before ECT assessed by ^18^F-FDG-PET/CT, and after 8 ECT sessions, hypometabolism revealed significant improvements in the left parietal cortex, the left temporal/occipital cortex, and bilateral frontal lobe. These improvements in brain glucose hypometabolism levels may represent a neurobiological mechanism of ECT for psychiatric disorders. However, inconsistent results were also reported by Reininghaus et al. (119). They used FDG-PET to assess changes of brain glucose metabolism before and after ECT in patients with depression and found the patients did not have significant changes of glucose metabolism levels before and after ECT; therefore, they did not believe that FDG-PET could assess changes in brain function after ECT.

### Arterial spin labeling

In recent years, the development of magnetic arterial spin-labeled perfusion fMRI (ASL-fMRI) has provided a new method to study CBF. Like PET and SPECT, ASL-fMRI can explore CBF, and without radiation. Now ASL-fMRI has been widely used to study the neural mechanisms of depression, but there are few ASL-fMRI studies on ECT. Shi et al. (120) focused on ASL differences in depressed patients by frontotemporal ECT and found significantly reduced rCBF in the bilateral frontal lobes before ECT, and after ECT, decreased rCBF was found in the left amygdala, parahippocampal gyrus, olfactory cortex, right occipital lobe, while increased rCBF was found in the bilateral frontal lobe. More studies using ASL-fMRI mainly focused on alterations in the hippocampus. Recently, Leaver et al. (121) tried to predict the clinical outcome of ECT measured by ASL, and found lower whole-brain CBF levels in the patient group at baseline predicted a better ECT outcome, while after acute treatment of ECT and 4 weeks follow-up, it showed elevated rCBF in the right anterior hippocampus, regardless of clinical outcome. On the other hand, this study found an elevated CBF in the dorsal thalamus as well as in the motor cortex and a decreased CBF in the frontotemporal areas in patients with a clinical response to ECT. Another study focused on the difference in cerebral perfusion in the hippocampus between responding group and non-responding group after ECT assessed by ASL and found that patients who did not respond to ECT showed increased CBF in the bilaterally anterior hippocampus, while patients who responded to ECT showed increased CBF in the bilateral posterior part of hippocampus (122). However, in a recent study, Bracht et al. (123) also focused on the CBF changed in the hippocampus, and found CBF increase in the hippocampus were observed in the ECT-group but not in treatment response group, showing that CBF in the hippocampus was not associated with antidepressant response.

## Conclusion

The development of brain imaging provide an important approach to the study of depression and ECT, Table 1 gives a summary of relatively consistent findings. However, there are still some limitations, and also should consider the directions for future research, mainly in the following aspects: (1) there are many choices of statistical methods, together with the different subjects, different MRI equipment parameters, resulted in a large number of inconsistent findings, so future research with more samples in multi-center is needed; (2) there are more studies focusing on the efficacy of ECT, but fewer studies on its side effects, such as cognitive impairment or delirium after ECT, and this is needed to be further investigated; (3) the efficacy of ECT may be related to multiple mechanisms, but there are few animal MRI studies on ECT, and it is difficult to study the mechanism, so in the future, more animal studies on ECT is needed.

**Table 1:** Relatively consistent findings in neuroimaging study after ECT.

sMRI: (1) gray matter volume: increase in hippocampus and amygdala; (2) cortical thickness: changes in temporal gyrus, anterior cingulate gyrus
DTI: White matter Alterations in microstructure
fMRI: FC/ReHo/ALFF/fALFF Changes in cingulate cortex and prefrontal cortex
MRS: Gln/Glx/GABA/NAA/Cho No consistent changes
PET: Neurotransmitters/glucose metabolism Changes in frontal regions
ASL: CBF Changes in the hippocampus

## Author contributions

XC: investigation, methodology, writing—original draft, and writing—review and editing. HY: conceptualization, investigation, methodology, supervision, and writing—review and editing. L-BC: conceptualization, investigation, methodology, project administration, supervision, and writing—review and editing. XL: conceptualization, investigation, methodology, project administration, resources, supervision, writing—original draft, and writing—review and editing. All authors contributed to the article and approved the submitted version.

## Conflict of interest

The authors declare that the research was conducted in the absence of any commercial or financial relationships that could be construed as a potential conflict of interest.

## Publisher’s note

All claims expressed in this article are solely those of the authors and do not necessarily represent those of their affiliated organizations, or those of the publisher, the editors and the reviewers. Any product that may be evaluated in this article, or claim that may be made by its manufacturer, is not guaranteed or endorsed by the publisher.
